# Supplementary material for: Piezoelectricity in chalcogenide perovskites
Source: Nat Commun. 2024 Jul 9;15:5768. doi: 10.1038/s41467-024-50130-5 (PMC11233625; doi:10.1038/s41467-024-50130-5)
Supplement: Supplementary file 1 — Supplementary Information [file 41467_2024_50130_MOESM1_ESM.pdf]

Supporting Information

Piezoelectricity in Chalcogenide Perovskites

Sk Shamim Hasan Abir[1*], Shyam Sharma[1*], Prince Sharma[2], Surya Karla[3], Ganesh Balasubramanian[4], Johnson Samuel[1#] and Nikhil Koratkar[1,5#]

[1]Department of Mechanical, Aerospace and Nuclear Engineering, Rensselaer Polytechnic Institute, Troy, NY, 12180, USA.

[2]Department of Mechanical Engineering and Mechanics, Lehigh University, Bethlehem, PA 18015, USA.

[3]Howard P. Isermann Department of Chemical and Biological Engineering and Center for Biotechnology and Interdisciplinary Studies, Rensselaer Polytechnic Institute, Troy, NY, 12180, USA

[4]Department of Mechanical and Industrial Engineering, University of New Haven, West Haven, CT, 06516, USA.

[5]Department of Materials Science and Engineering, Rensselaer Polytechnic Institute, Troy, NY, 12180, USA.

* These authors contributed equally to this work

[#]Correspondence to be addressed to J.S. (samuej2@rpi.edu) and N.K. (koratn@rpi.edu)

Supplementary Text

We performed detailed first principles calculations to study the role of flexoelectricity in $BaZrS_3$. Flexoelectricity is a 4th order tensor described as the polarization response to strain gradient, where $P_i = f_{ijkl}\frac{\partial \varepsilon_{kl}}{\partial x_j}$ is the polarization induced by the strain gradient, f_{ijkl} are flexoelectric tensors and $\frac{\partial \varepsilon_{kl}}{\partial x_j}$ is the strain gradient. To study flexoelectricity in $BaZrS_3$, first-principles calculations are performed with the Vienna ab initio Simulation Package (VASP).[1] Atomic interactions are described using the Projector Augmented Wave (PAW) method.[2] The Perdew-Burke-Ernzerhof (PBE) generalized gradient approximation (GGA) functional is employed to account for exchange-correlation effects.[3] A planewave expansion with an energy cutoff of 520 eV is utilized.[4] The Brillouin zone is sampled using a Monkhorst-Pack k-points mesh grid with a precision level of 0.03.[5]

To circumvent the issue of periodic boundary conditions in a supercell with non-uniform strain, we construct an accordion supercell where strain gradient is sinusoidal in nature and hence preserves periodicity of the boundaries – this same approach has been used in Ref. 6–8. Consequently, the strain distribution adheres to a cosine variation, while displacements follow a sinusoidal curve: $\delta(z) = \varepsilon_{max} * \frac{h*\sin({}^{2\pi z}/_{h})}{2\pi}$; $\varepsilon(z) = \varepsilon_{max} * \cos({}^{2\pi z}/_{h})$; and $\frac{\partial \varepsilon}{\partial x} = -\varepsilon_{max} * \frac{2\pi*\sin({}^{2\pi z}/_{h})}{h}$. Here $\delta(z)$ is the atomic displacement in the longitudinal direction, ε_{max} is the maximum strain, z is the atom's coordinate in longitudinal direction and h is the total length of the supercell in the longitudinal direction.

Fig. S14 shows the variation of displacement, strain, and strain gradient from our calculations on a 40.16 Å long supercell. The initiation of flexoelectricity is based on the breaking of inversion symmetry by virtue of strain gradient, while piezoelectricity is generated due to a uniform strain throughout. Hence for piezoelectricity one unit cell is sufficient to mimic the entire system, while in case of flexoelectricity one must consider a supercell large enough to enable a converged displacement profile. Here, we demonstrate a converged atomic displacement within a 40.16 Å long supercell, consistent with previous calculations.[6-8]

We apply different magnitudes of strain gradients on the relaxed supercells (Ba positions constrained) and calculate polarization at h/4 where the strain is zero and strain gradient is maximum, hence eliminating piezoelectric contributions. Calculations were performed for strain gradients up to $\pm 4 \times 10^7\ m^{-1}$ (Fig. S15) where polarization follows a linear relationship with strain, akin to the approach by Shin et al.[8]

The longitudinal flexoelectric coefficient, derived from the slope of the curve in Fig. S15, is very small (~0.0193 nC/m). This value, determined at the point where strain is zero and strain gradient is maximum, indicates a negligible contribution of flexoelectricity to polarization generation or the emergence of a non-zero dipole moment during deformation. Consequently, this result suggests that the role of flexoelectricity in generation of polarization or non-zero dipole moment under deformation is minimal for $BaZrS_3$.

Supplementary Tables

Material	d_{ij}^{max} (pC/N)	Material	d_{ij}^{max} (pC/N)
Zinc oxide (ZnO)	12.3	Li-modified lead-free $(Na,K)NbO_3$ (KNN-CT)	241
Bismuth ferrite (BFO)	37	$(K,Na)NbO_3$-$LiSbO_3$ (LNKN)	317
Lead magnesium niobate - lead titanate (PMN-PT)	-74	$(K,Na,Li)(Nb,Ta,Sb)O_3$ (KNLNTS)	416
Polycaprolactone (PCL)	4	$(K,Na)(Nb,Ta)O_3$ (KNNT)	390
Lead zirconate titanate (PZT-5H)	593	Doped $PbTiO_3$ (PZ34)	46
Lead zirconate titanate (PZT-5K)	870	Cadmium sulfide (CdS)	10.65
$PbZn_{1/3}Nb_{2/3}O_3$ - 7%$PbTiO_3$ (PZN - 7% PT)	2400	$BaTiO_3$ (BT)	270
Sodium–Potassium Niobate (ANSZ)	295	$Pb(Zn_{1/3}Nb_{2/3})_{0.92}Ti_{0.08}O_3$ (PZNT8)	2500
Polyvinylidene fluoride (PVDF)	-27.1	$LiTiO_3$ (LTO)	26

Table S1 | Commonly observed piezoelectric materials. Highest performing materials all contain lead and are highlighted in yellow (https://en.wikipedia.org/wiki/List_of_piezoelectric_materials).

$CaTiS_3$	$SrTiS_3$	$BaTiS_3$
$CaZrS_3$	$SrZrS_3$	$BaZrS_3$
$CaHfS_3$	$SrHfS_3$	$BaHfS_3$
$CaTiSe_3$	$SrTiSe_3$	$BaTiSe_3$
$CaZrSe_3$	$SrZrSe_3$	$BaZrSe_3$
$CaHfSe_3$	$SrHfSe_3$	$BaHfSe_3$

Table S2 | List of possible chalcogenide perovskite compounds

Supplementary Figures

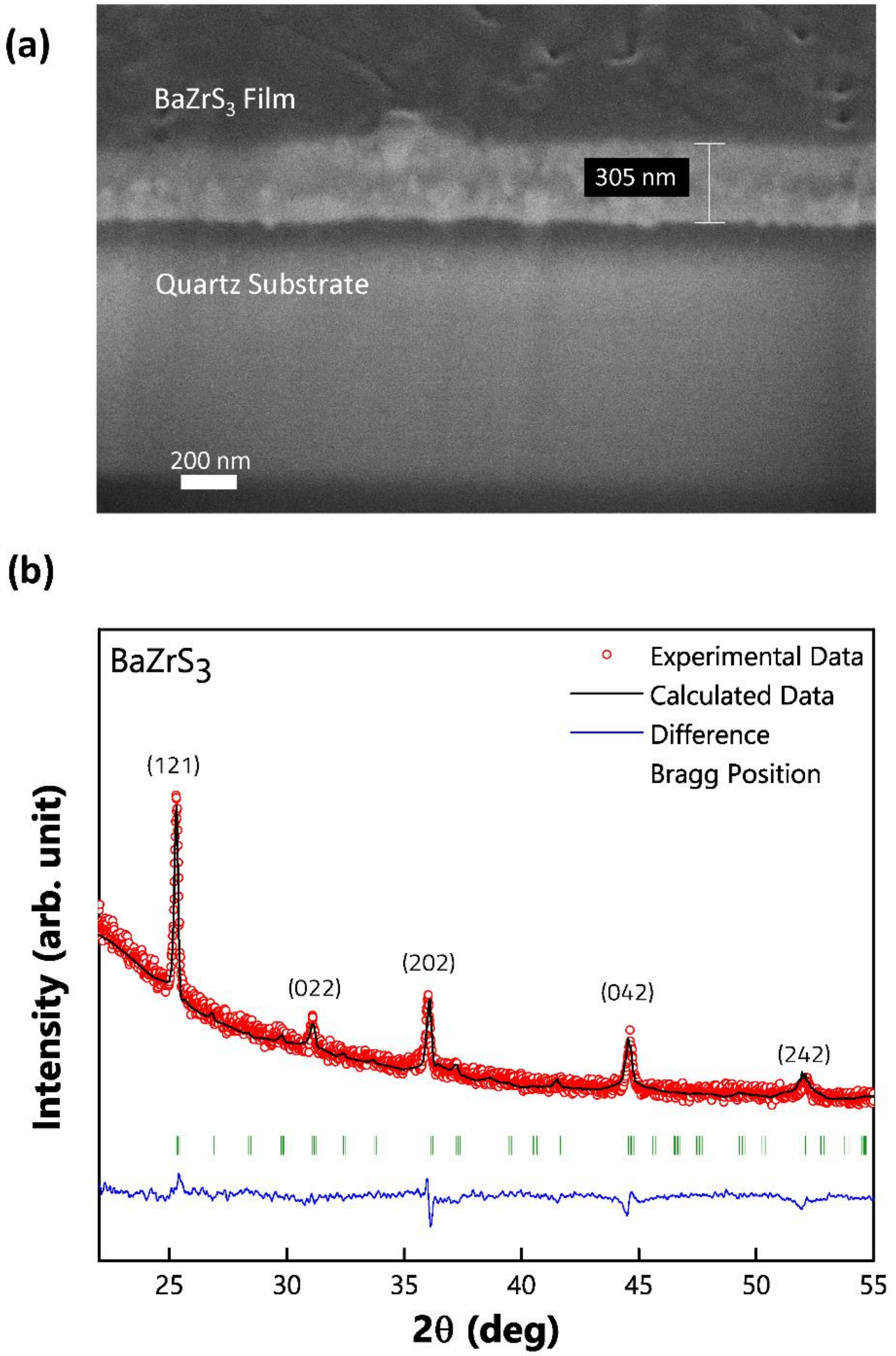

Figure S1 | (a) Cross-sectional scanning electron microscopy (SEM) image of a typical $BaZrS_3$ thin film over quartz. (b) XRD and Rietveld refined profile of $BaZrS_3$ film over quartz substrate.

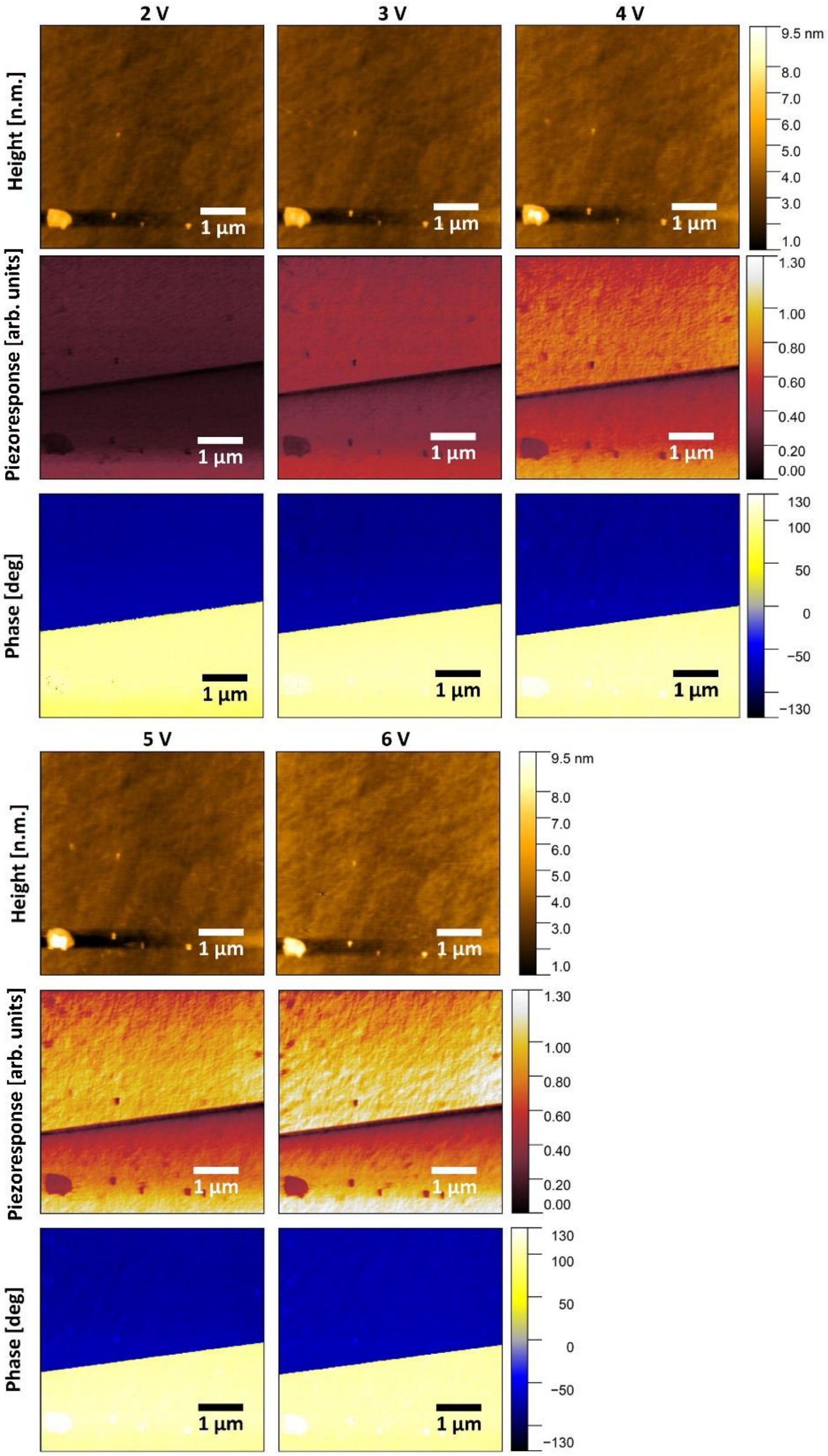

Figure S2 ǀ Periodically poled lithium niobate (PPLN) vertical piezoresponse force microscopy (PFM) height, amplitude and phase images at voltages of 2-6 V.

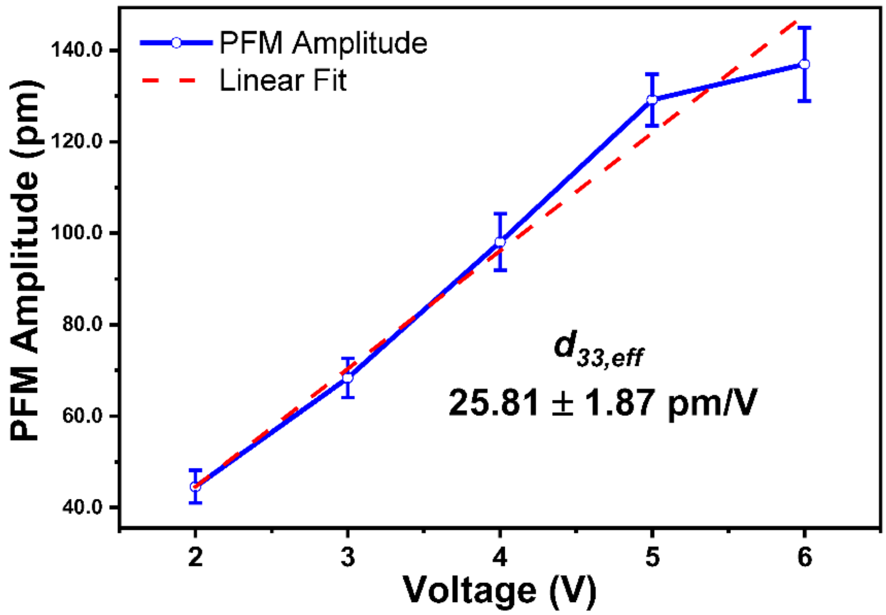

Figure S3 | PFM amplitude as a function of applied voltage showing the measured effective piezoelectric charge coefficient ($d_{33,eff}$) for periodically poled lithium niobate (PPLN) sample.

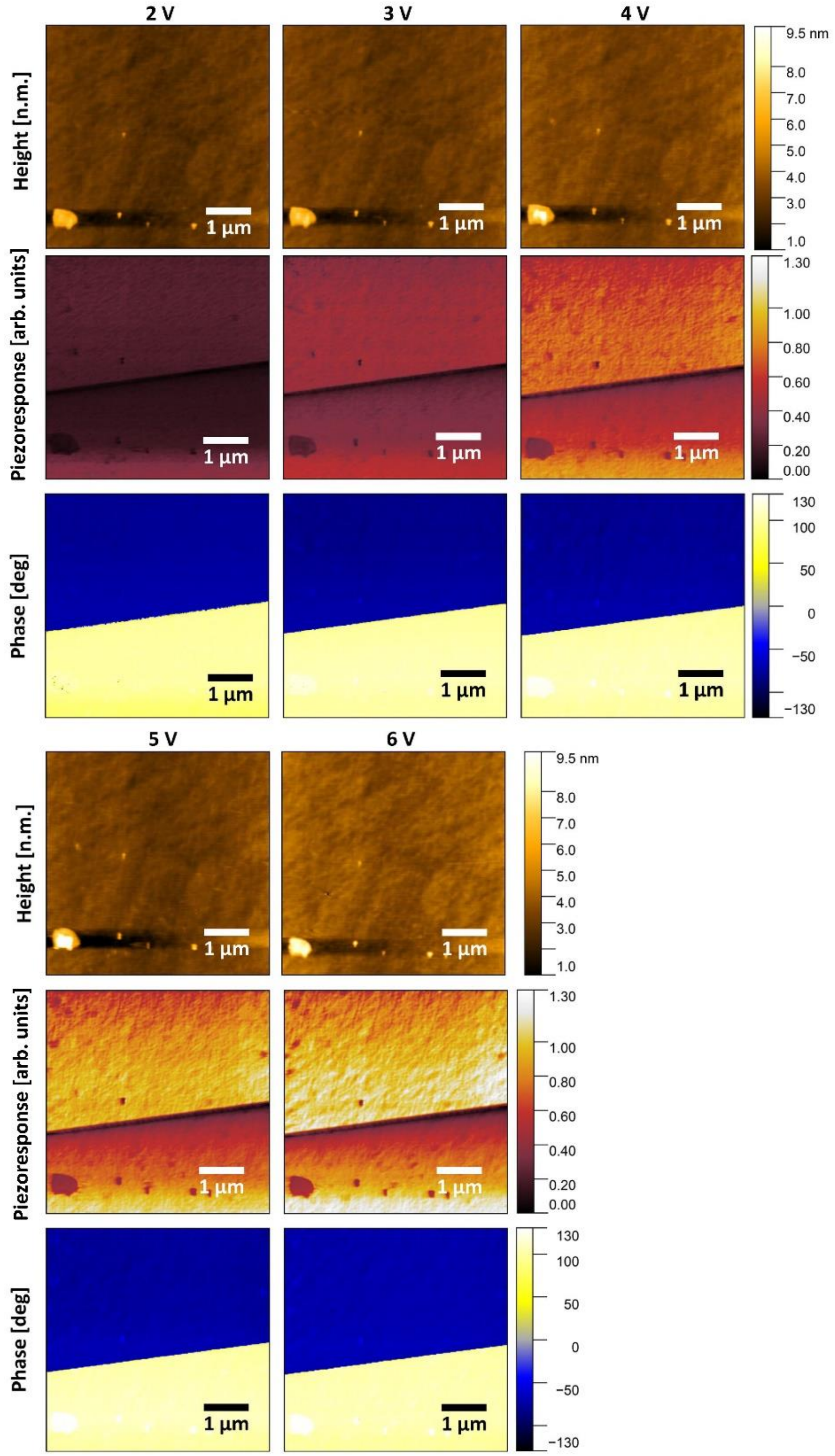

Figure S4 | $BaZrS_3$ vertical piezoresponse force microscopy (PFM) height, amplitude and phase images at voltages of 2-6 V.

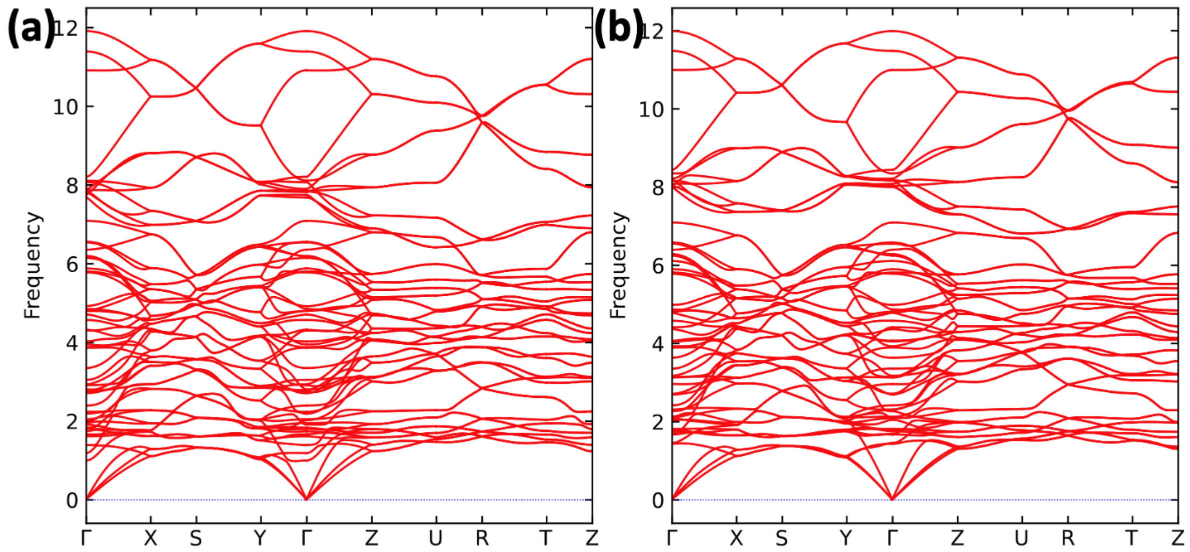

Figure S5 | Phonon band structure for $BaZrS_3$ (a) without including random displacments and (b) including random displacements. These results suggest that the structure is dynamically stable and highlights absence of imaginary phonons.

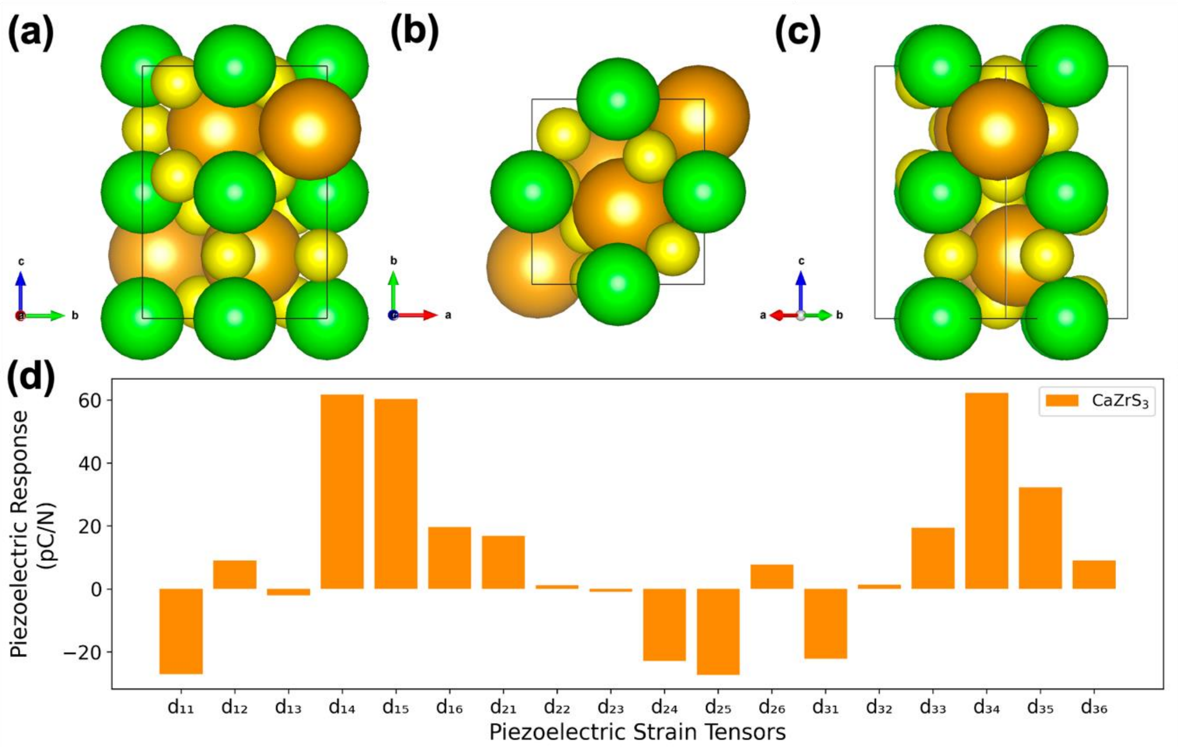

Figure S6 | (a-c) Projections of $CaZrS_3$ (Pnma) orthorhombic unit cell and (d) shows bar plots for piezoelectric strain tensors ($\boldsymbol{d_{ij}}$) for $CaZrS_3$.

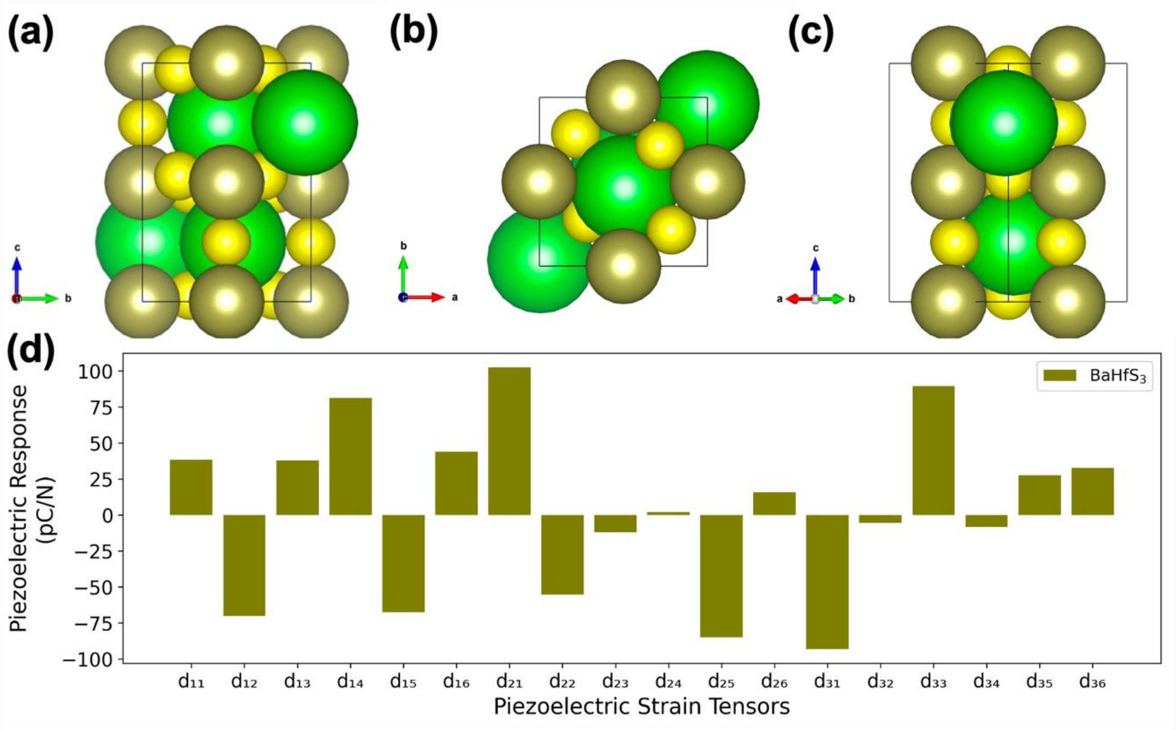

Figure S7 | (a-c) Projections of $BaHfS_3$ (Pnma) orthorhombic unit cell and (d) shows bar plots for piezoelectric strain tensors ($\boldsymbol{d_{ij}}$) for $BaHfS_3$.

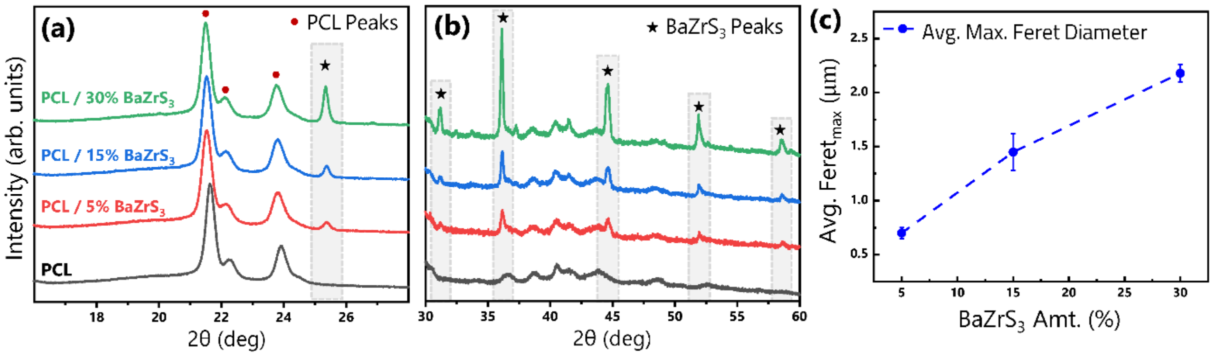

Figure S8 | $BaZrS_3$ encapsulated PCL film characterization: (a-b) XRD of the PCL-$BaZrS_3$ composite films for various weight fraction of $BaZrS_3$ additives. (c) Analysis of average particle size with increasing $BaZrS_3$ loading from maximum Feret diameters of the elemental sulfur maps.

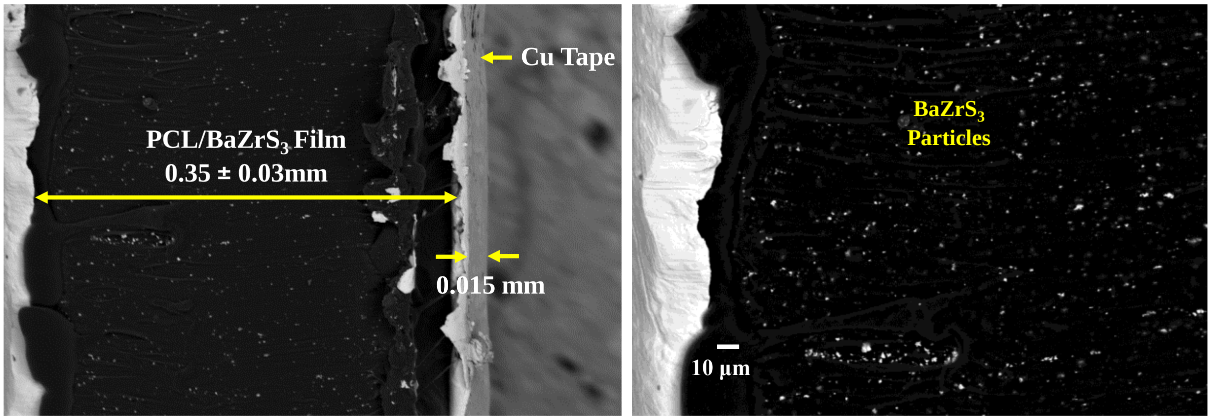

Figure S9 | Backscattered electron microscopy cross-section images of a PCL/$BaZrS_3$ composite film sandwiched between two Cu tapes that serve as electrodes. The $BaZrS_3$ loading fraction in this case is ~10 wt%.

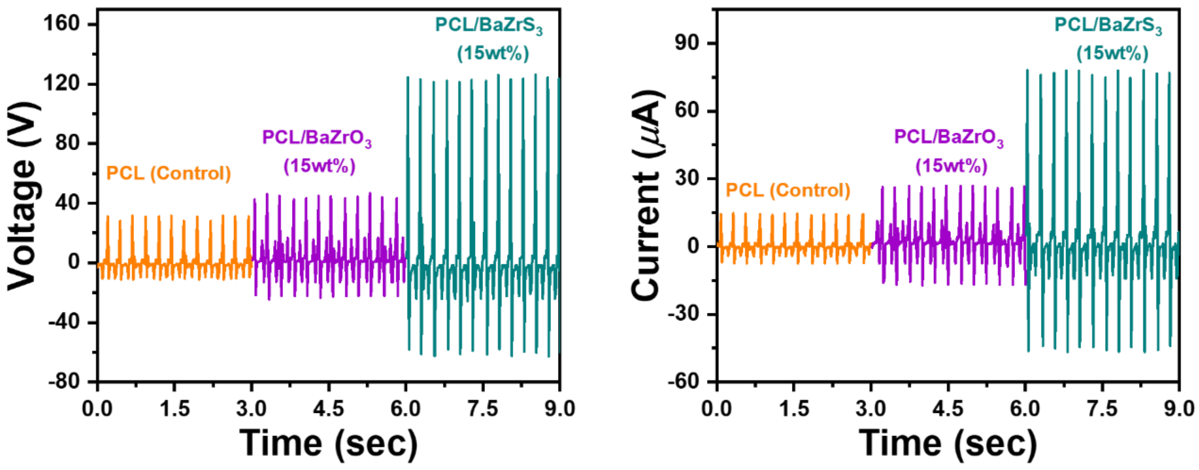

Figure S10 | Comparison of $\boldsymbol{V_{OC}}$ and $\boldsymbol{I_{SC}}$ at ~30 PSI pressure and ~4 Hz load frequency for the PCL(Control), and the PCL/$BaZrO_3$(15.0 wt%) and PCL/$BaZrS_3$(15.0 wt%) composites.

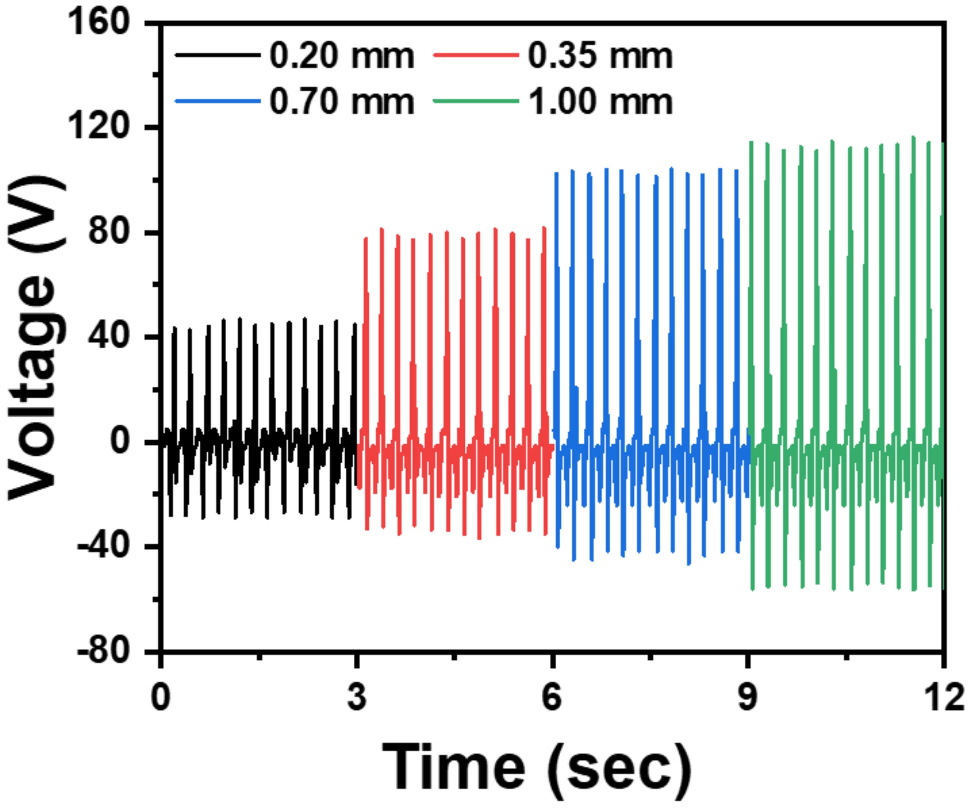

Figure S11 | Open circuit voltage response of PCL/$BaZrS_3$ (~10 wt%) composite film at ~0.20, ~0.35, ~0.70, and ~1.0 mm film thickness.

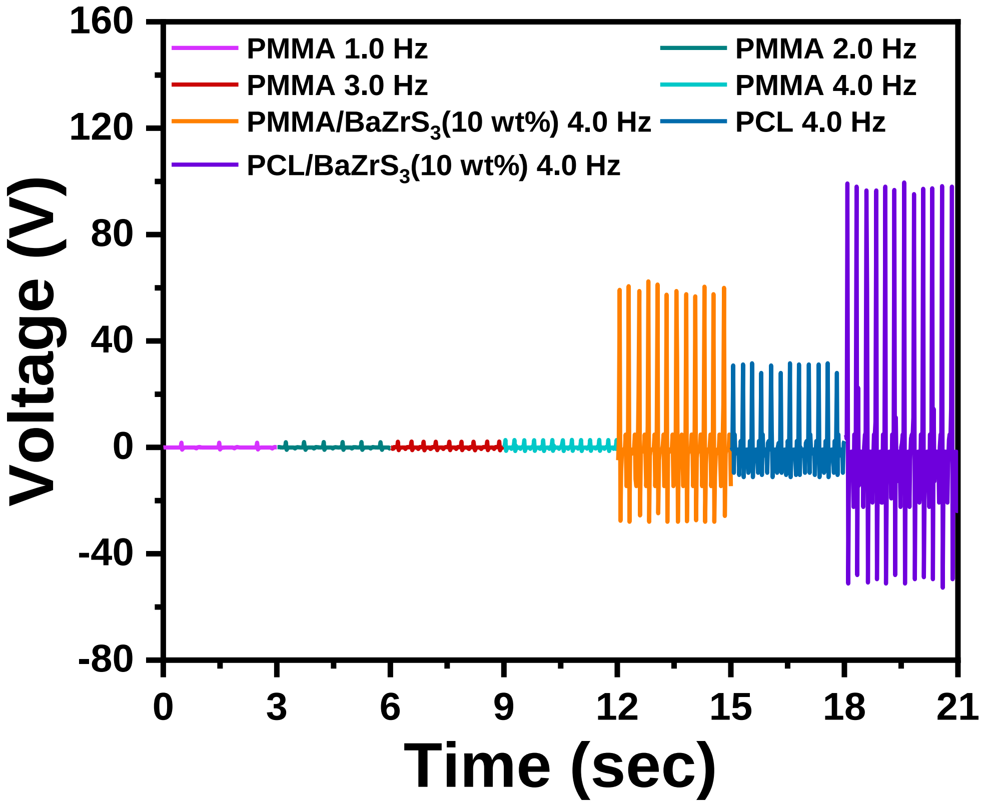

Figure S12 | Open circuit voltage response of PMMA polymeric film for 1.0–4.0 Hz load frequency, PMMA/$BaZrS_3$ (~10 wt%) composite film, PCL film, and PCL/$BaZrS_3$ (~10 wt%) composite film for ~4.0 Hz load frequency. All samples were tested at ~30 PSI applied pressure.

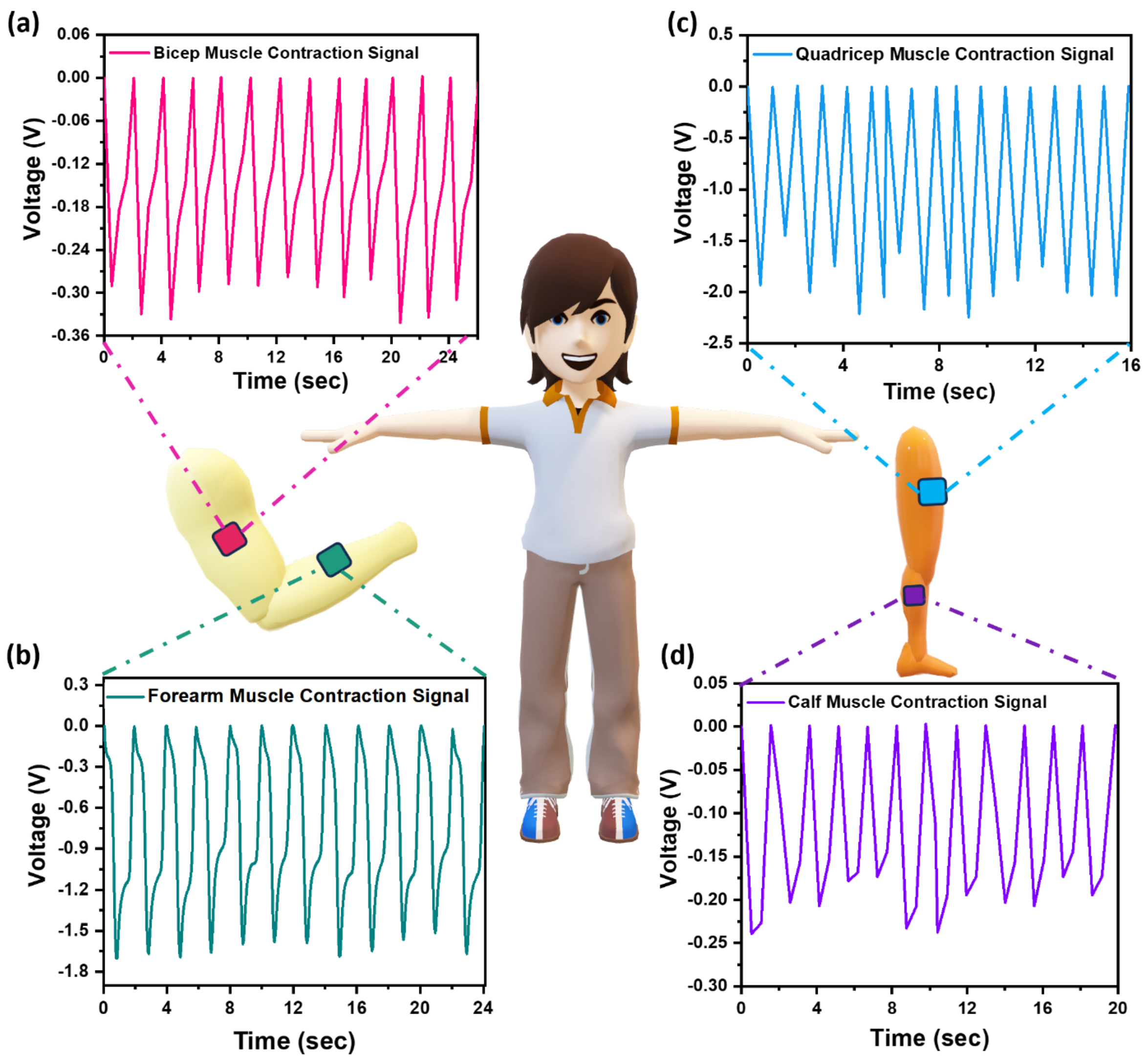

Figure S13 | Sensing performance of PCL-$BaZrS_3$(15wt%) composite: (a) Bicep and (b) forearm muscle flexing with 5 lb load weight lift. (c) Quadricep and (d) calf muscle flexing during regular leg up-down movement.

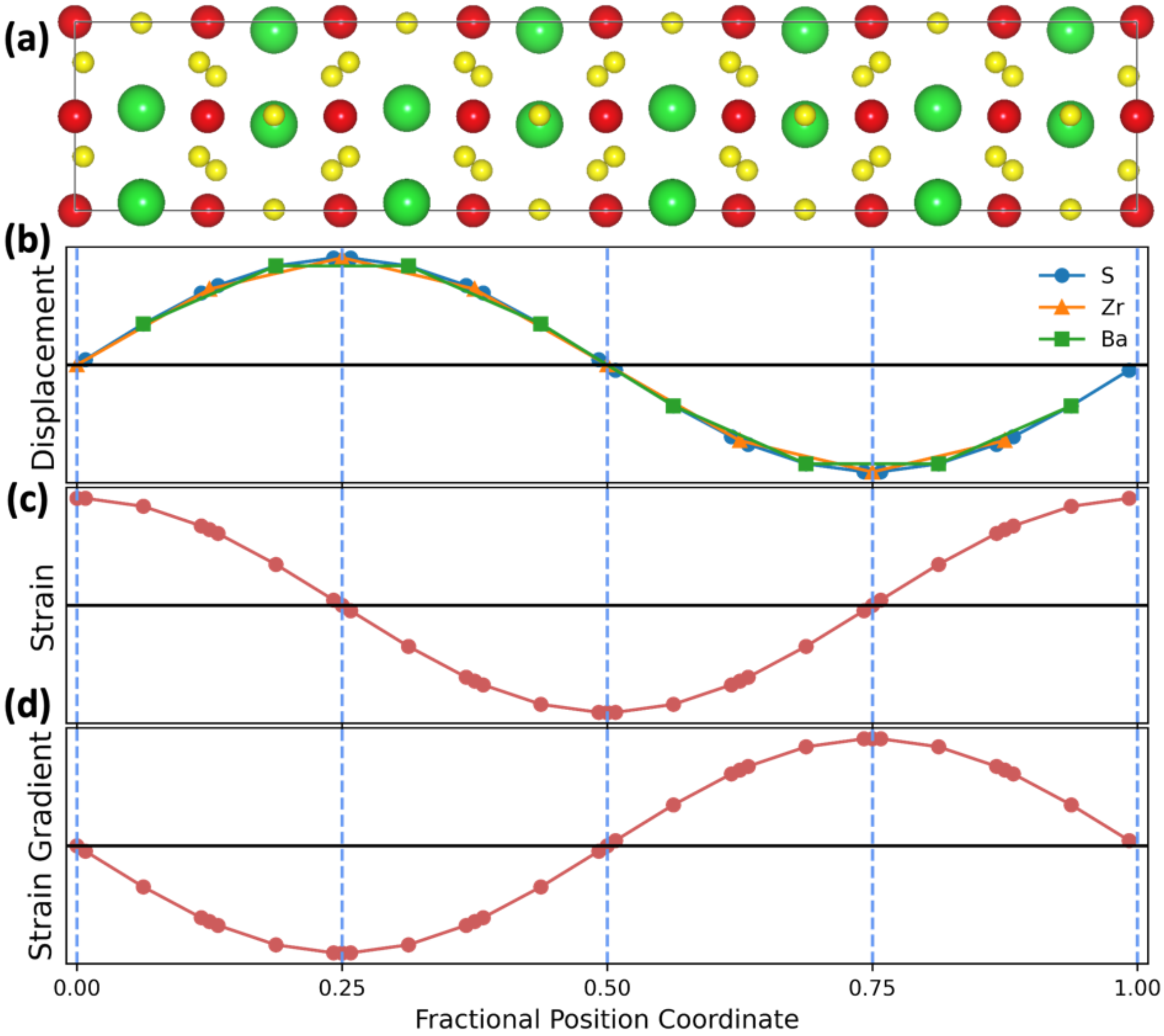

Figure S14 | Displacement and strain patterns within (a) $BaZrS_3$ supercell of 40.16 Å length, illustrating (b) sinusoidal atomic displacements, (c) strain variation assuming a cosine profile, and (d) sinusoidal profile for strain gradient within the supercell with fractional position coordinates.

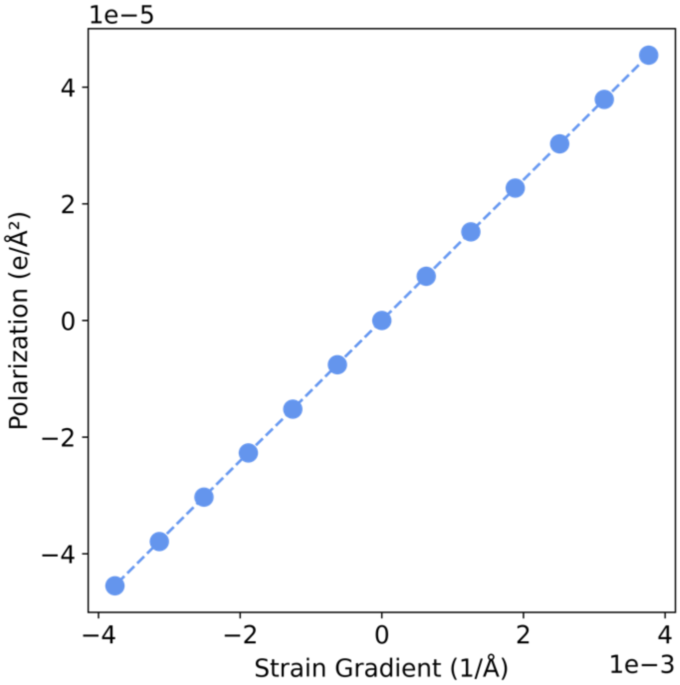

Figure S15 | Polarization as a function of strain gradient. The slope of the curve is the longitudinal flexoelectric coefficient.

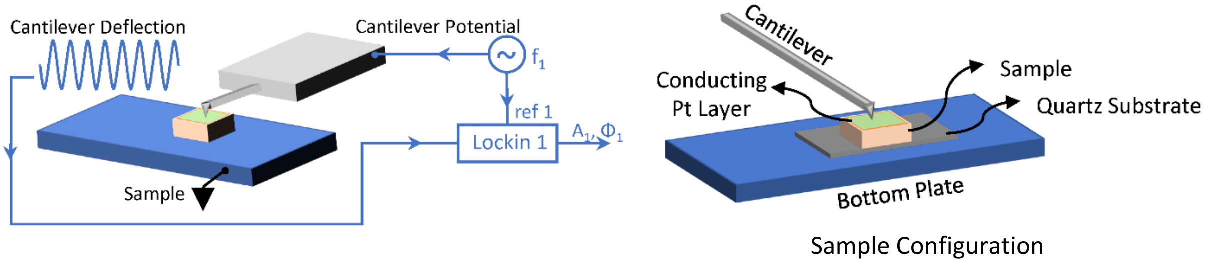

Figure S16 | (Left) Schematic diagram of Single Frequency PFM, (Right) Configuration used in our tests.

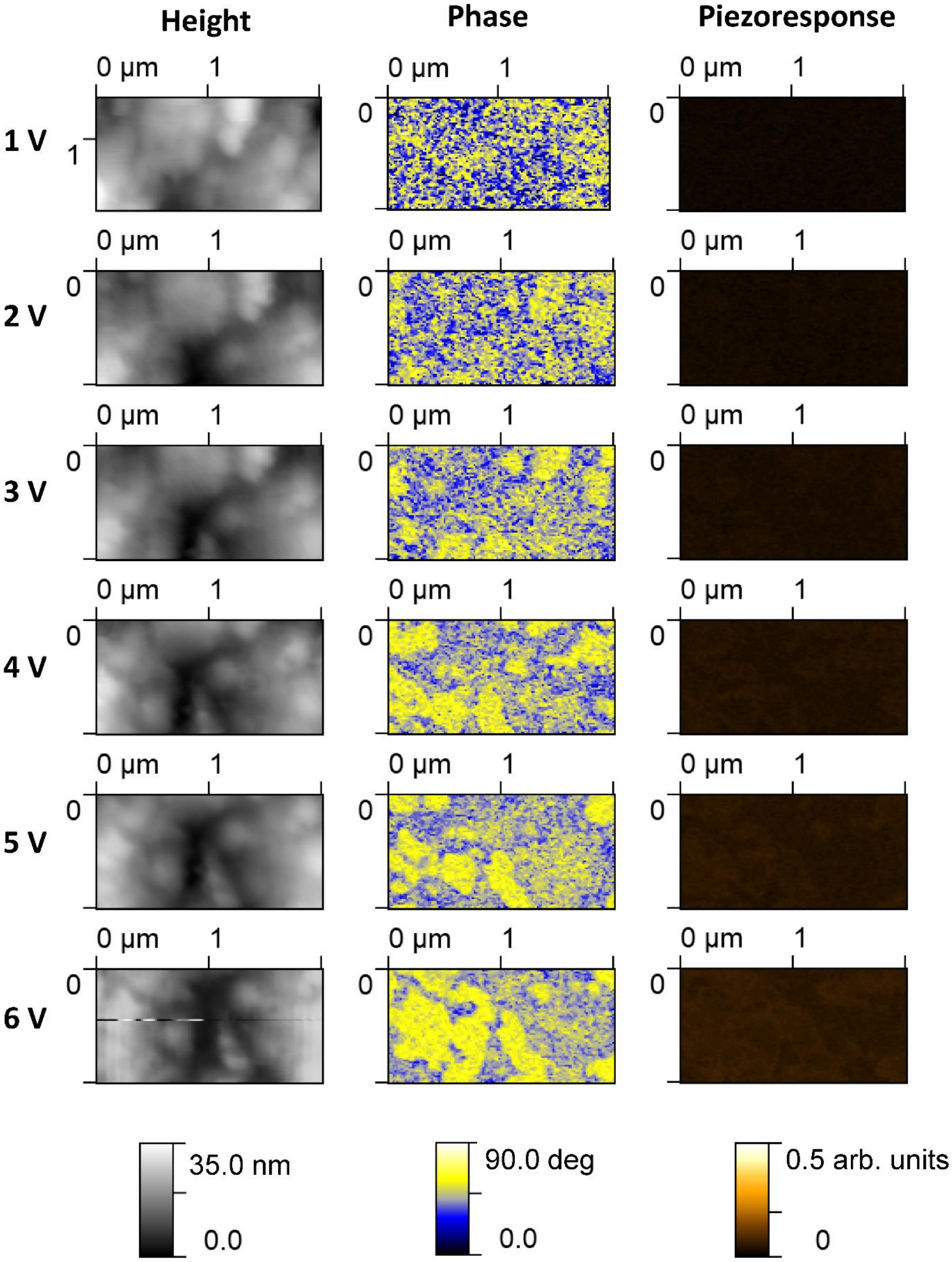

Figure S17 | $BaZrO_3$ thin film over quartz vertical piezoresponse force microscopy (PFM) height, phase and amplitude images at voltages of 1-6 V.

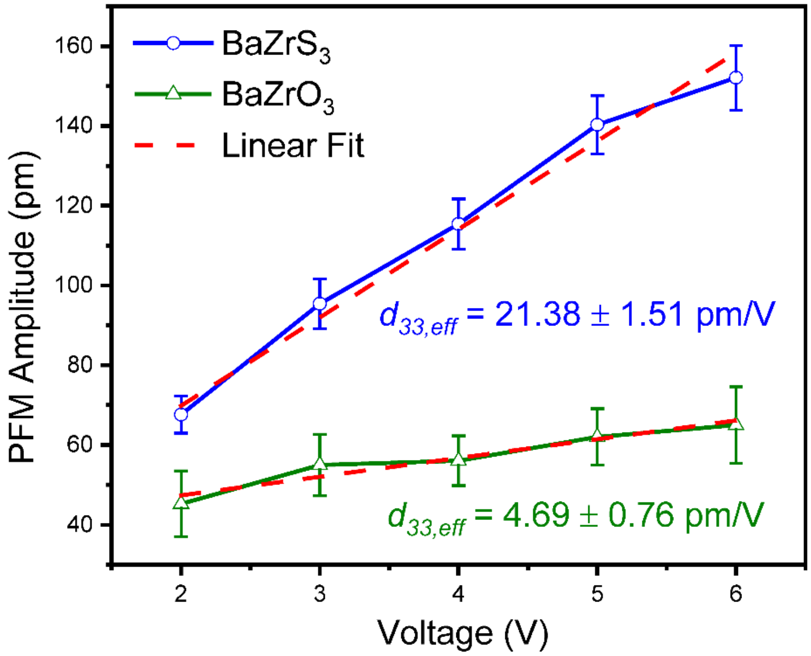

Figure S18 | PFM amplitude as a function of applied voltage showing the measured effective piezoelectric charge coefficient ($d_{33,\,eff}$) for $BaZrO_3$ vs $BaZrS_3$.

Supplementary References

1. Hafner, J. Ab-initio simulations of materials using VASP: Density-functional theory and beyond, *J. Comput. Chem.* **29**, 2044–2078 (2008).
2. Kresse, G., Joubert, D. From ultrasoft pseudopotentials to the projector augmented-wave method, *Phys. Rev. B.* **59**, 1758 (1999).
3. Perdew, J. P., Burke, K., Ernzerhof, M. Generalized Gradient Approximation Made Simple, *Phys. Rev. Lett.* **77**, 3865 (1996).
4. Methfessel, M., Paxton, A. T. High-precision sampling for Brillouin-zone integration in metals, *Phys. Rev. B* **40**, 3616 (1989).
5. Monkhorst, H. J., Pack, J. D. Special points for Brillouin-zone integrations, *Phys. Rev. B* **13** 5188, (1976).
6. Hong, J., Catalan, G., Scott, J. F., Artacho, E. The flexoelectricity of barium and strontium titanates from first principles, *Journal of Physics: Condensed Matter* **22**, 112201 (2010)
7. Plymill, A., Xu, H. Flexoelectricity in $AtiO_3$ (A = Sr, Ba, Pb) perovskite oxide superlattices from density functional theory, *J. Appl. Phys.* **123**, 144101 (2018).
8. Shin, Y.-H., Ali, A., Kim, H.J., Kim, T.H. Theoretical estimation of longitudinal flexoelectric response in polar perovskite oxides, *Res. Sq.* (2022); https://doi.org/10.21203/rs.3.rs-2201998/v1
